# Triple Cultures Increase the Diagnostic Sensitivity of Mycobacterial Tuberculosis Empyema

**DOI:** 10.1155/2017/4362804

**Published:** 2017-09-05

**Authors:** Kingsfield Ong, Keerthi Rajapaksha, Chin Siang Ong, Ali Akbar Fazuludeen, Aneez Dokeu Basheer Ahmed

**Affiliations:** ^1^Division of Thoracic Surgery, Department of General Surgery, Tan Tock Seng Hospital, Singapore; ^2^Johns Hopkins School of Medicine, Baltimore, MD, USA

## Abstract

**Background:**

There is a continuous debate on the appropriate diagnostic approach and surgical management of mycobacterial empyema, with widely varied diagnostic practices and surgical outcomes. The aim of this study is to highlight the diagnostic approach and clinical features of patients who required surgical intervention for mycobacterial empyema.

**Methods:**

We performed a 5-year retrospective cohort study of all patients with mycobacterial empyema requiring surgery in a single institution from November 2009 to November 2014.

**Results:**

Eighteen patients (15 males and 3 females, median age 48.5 years) required surgery. Seventeen patients required decortication via posterolateral thoracotomy and one patient underwent video-assisted thoracic surgery drainage and pleural debridement. Prolonged air leak was the commonest surgical complication (50%, *n* = 9). 94.4% (*n* = 17) had necrotizing granulomatous inflammation on histological examination. The sensitivity of mycobacterium smear and culture ranged between 12.5% and 75% for pleural tissue, sputum, and pleural fluid individually. The combination of all 3 samples increased the diagnostic yield to 100%.

**Conclusion:**

With the implementation of pleural tissue culture at surgery, the novel combination of sputum, pleural fluid, and pleural tissue culture provides excellent diagnostic yield.

## 1. Introduction

From the days of Hippocrates to Robert Koch, followed by Camille Guerin and progressing to an era of potent antimycobacterial agents, mycobacterial infection is still regarded as a serious medical condition. Mycobacterial empyema is a less common but severe complication of the mycobacterial disease [1], with high morbidity and mortality in the absence of timely medical and surgical intervention [2–4]. It usually manifests late, after failing medical treatment, as an organized empyema (American Thoracic Society classification) [5].

However, there is no consensus on the appropriate diagnostic approach and surgical management of mycobacterial empyema, with widely varied diagnostic practices and surgical outcomes documented in previous literature. In fact, about 40% of these cases are culture negative [1, 6]. Furthermore, guidelines for the treatment of mycobacterial empyema are pointing towards pathogen-specific therapy [6]. Hence, the isolation of the pathogen and the determination of its drug sensitivities are crucial, especially in patients with mycobacterial empyema where prompt diagnosis is imperative to its management. The purpose of this study is to review the diagnostic approach and clinical features of patients who underwent surgical intervention for mycobacterial empyema over a 5-year period in our institution and examine if we can improve the diagnostic issues with novel combination testing.

## 2. Methods

We performed a 5-year retrospective cohort study of mycobacterial empyema patients who required surgical intervention at the Division of Thoracic Surgery, Tan Tock Seng Hospital, Singapore, during the period of November 2009 to November 2014.

We routinely proceeded with the following investigations, which included AFB smear and culture of sputum (2 to 3 sets), pleural fluid and pleural tissue, and histology of pleural tissue, in all patients suspected of mycobacterial empyema. The criteria for the diagnosis of mycobacterial empyema were met if either of the following was positive: (1) positive acid fast bacilli (AFB) smear or culture from sputum or (2) positive AFB smear or culture from pleural fluid or (3) positive AFB smear or culture from pleural tissue or (4) necrotizing granulomatous inflammation on histological examination. There were no nondiagnostic procedures performed prior to surgery. In addition, the respiratory team routinely performed a pleural tap or chest tube insertion and sent off one set of pleural fluid AFB smears and cultures before referring the patient for surgery.

The criteria for surgical intervention were the presence of loculated empyema with thickened pleura on computed tomography (CT) scan of the chest, which was noted in 17 out of 18 patients. One patient had persistent fever with effusion noted on chest X-ray despite antibiotics and a chest drain* in situ*. Hence, the decision for surgery was made to address source control. All patients underwent general anesthesia and double lumen endotracheal intubation, with isolation of the affected lung. Postoperatively, patients were monitored in the surgical intensive care unit or high dependency unit.

Medical reports were reviewed to obtain the following data: demographics, clinical symptoms, comorbidities, preoperative investigations, microbiology, histology, surgical outcomes, and complications. Radiological reports of the contrast-enhanced computed tomography of the thorax were reviewed together with images to identify key radiological features that were present in this cohort of patients. Clinical data recorded were presented as median (range) and frequencies in percentages. The sensitivities of the diagnostic tests were recorded as percentages.

This study received approval from the Domain Specific Research Board, National Health Care Group (Singapore), with waiver of patient consent.

## 3. Results

### 3.1. General Characteristics (Table 1)

Eighteen patients (15 males, 3 females) with a median age of 48.5 years (range 27–73 years) required surgical intervention for mycobacterial empyema. Comorbidities included hypoalbuminemia (albumin < 35 g/L) (83.3%), diabetes mellitus (22.2%), coronary artery disease (5.6%), chronic obstructive pulmonary disease (5.6%), chronic renal failure requiring dialysis (5.6%), and immunocompromised state (5.6%). The ratio of the side of empyema (right to left) was equal, at 1 : 1.

### 3.2. Clinical Characteristics (Table 2)

The majority of patients presented with cough (88.9%) followed by fever (50%). Shortness of breath (38.9%) and chest pain (11.1%) were the other common presenting symptoms. One patient presented with a cold abscess in the chest wall. CT scan of the thorax was performed in 94.4% (*n* = 17) of patients prior to surgery. All the patients who had preoperative CT scans had loculated empyema with thickened pleura. Other notable features included tree-in-bud appearance, hydropneumothorax, flattened chest wall, bronchieactasis, obliteration of bronchus, calcified pleura, cavitary parenchymal lesions, and mediastinal lymphadenopathy.

### 3.3. Microbiological Analysis (Table 3)

*Mycobacterium tuberculosis* empyema was diagnosed in all (*n* = 18) of the patients. Positive AFB smears in sputum, intraoperatively pleural fluid, and pleural tissues were noted in 23.5%, 12.5%, and 25% of patients, respectively. By combining the three specimens, the sensitivity increased to 44.4%. Positive mycobacterium culture in sputum, intraoperative pleural fluid, and pleural tissues were found in 41.1%, 41.1%, and 75% of patients, respectively. The combination of sputum, intraoperatively pleural fluid, and pleural tissue culture increased the diagnostic sensitivity to 94.1%. One patient did not have the pleural tissue and fluid AFB smear investigated as it was inadvertently left out. In another patient, we did not send the pleural tissue for AFB culture as the preoperative pleural tissue AFB culture sent by the respiratory team was already positive.

Routinely 2 to 3 sets of sputum AFB smears and cultures were sent. The combination of the former with a single sample of intraoperative pleural fluid and tissue AFB smear and culture confers a diagnostic yield of 100%. One patient had a positive pleural fluid culture preoperatively from a chest drain and received 4 weeks of the standard first-line regime of rifampicin, isoniazid, ethambutol, and pyrazinamide antituberculosis treatment before surgical decortication. Therefore, we excluded this patient from the diagnostic yield analysis.

One patient had superimposed* Enterobacter* bacterial infection and was treated with a short course of intravenous piperacillin/tazobactam and discharged with oral levofloxacin. There were no patients found to have superimposed fungal infection.

94.4% (*n* = 17) were found to have necrotizing granulomatous inflammation on histological examination of pleural tissue.

### 3.4. Surgical Outcomes and Complications (Table 4)

Seventeen patients required decortication via posterolateral thoracotomy and one patient underwent video-assisted thoracic surgery drainage and pleural debridement (median operative time 112.5 minutes, range 55–175 minutes). The commonest surgical complication of prolonged air leak of more than 5 days was found in 50% (*n* = 9) of patients. Median chest tube duration was 5.5 days (range 3–56 days). Median hospital stay was 5.5 days (range 3–14 days). Failure of lung reexpansion was noted in 16.7% (*n* = 3) of patients at the 6th-month follow-up clinic review. None of the patients required any further reexploration or reintervention and the 30-day surgical mortality was zero.

## 4. Discussion

Mycobacterial empyema is a collection of pus in the pleural space seeded with mycobacterium. Studies postulate that it is a result of prolonged unresolved pleurisy caused by the release of mycobacterium from its containing lung cavity into the pleural space [3, 7, 8]. Infrequently, some are caused by direct hematogenous spread [3]. Although the global incidence of tuberculosis is on the decline with the widespread use of potent antituberculous [4] agents, there is a surge in the number of tuberculosis infections in immunocompromised patients with conditions such as HIV, cancer, and organ transplant [3, 4]. Furthermore, recent studies have demonstrated a substantial number of nontuberculous mycobacterial infections in the population and a rise in the incidence of this type of infection [9]. Unfortunately, diagnosis and treatment are equally tricky [9].

Many authors define tuberculous empyema as purulent effusion with the presence of large amounts of mycobacterium and believe that AFB smears and mycobacterial cultures are frequently positive [7, 10, 11]. However, studies have shown that this is not the case [3]. Likewise, in our study, the pleural fluid smear and culture were positive in only 12.5% and 41.4% of patients, respectively. We routinely send decorticated pleural tissue for culture and the sensitivity was 75%. The novel combination of sputum, pleural fluid, and pleural tissue culture increases the sensitivity to 100%. This practice will be particularly useful in numerous centers over the world where high-tech or expensive diagnostic tests are limited. Thus, we encourage the culture of pleural tissue for better diagnostic yield to facilitate pathogen-specific therapy.

Tuberculosis related mortality rates range from 11.4% to 43.6% [3]. In the past, treatment outcomes were abysmal in mycobacterial empyema patients [12]. Advances in thoracic surgery, thoracic anesthesia, and potent antituberculous medications have dramatically improved the outlook the disease, and hence we are able to report zero mortality in our series. Likewise, better outcomes have been demonstrated in recent literature [3, 4]. The age distribution in this study demonstrates a high preponderance amongst the middle aged, which is similar to previous studies [3, 4]. 77.8% (*n* = 14) of our patients were in a state of hypoalbuminemia, reflecting the indolent nature and chronicity of the disease. Cough, fever, and shortness of breath were the most common complaints in our cohort. Although prolonged air leak was a common complication postoperatively in this series, all resolved without further surgical intervention. The patients were usually discharged with a flutter bag and reviewed at the outpatient clinic.

Our study has its limitations, such as the small sample size and its retrospective nature. Our cohort of surgical patients may not be representative of patients with tuberculous pleural effusions. Nonetheless, as our diagnostic yield is similar to patients with tuberculous effusion [13], we postulate that triple cultures will increase the diagnostic yield in such patients as well.

We recognize that the diagnostic criteria for mycobacterial empyema are culture positivity for mycobacteria in sputum, pleural effusion, or pleural tissue. However, in clinical practice, sometimes only 2 cultures (e.g., sputum culture and pleural fluid culture) were performed. More importantly, we found that sending one set of mycobacterial cultures (sputum, pleural fluid, or pleural tissue) only provided a sensitivity of 41.1% to 75%, whereas if 3 sets of cultures (sputum, pleural fluid, and tissue) were performed, the combined sensitivity is 100%. Sending off all 3 cultures will allow the disease to be recognized and treated early. In addition, it allows the mycobacteria to be isolated for drug sensitivity testing. This is especially important with the rise in multidrug resistant tuberculosis.

In summary, with the integration of pleural tissue culture, the novel combination of sputum, pleural fluid, and pleural tissue culture provides excellent diagnostic yield as compared to other combination methods in previous studies. Timely surgical intervention for mycobacterial empyema is both safe and effective for the diagnosis and treatment of this disease.

## Figures and Tables

**Table 1:** Patient characteristics.

Characteristics	*n* (%)
Age^*∗*^	48.5 (27–73)
Male-to-female ratio	5 : 1
Hypertension	4 (22.2%)
Coronary artery disease	1 (5.6%)
Chronic obstructive pulmonary disease	1 (5.6%)
Diabetes	4 (22.2%)
Renal failure requiring dialysis	1 (5.6%)
Immunocompromised	1 (5.6%)
Hypoalbuminaemia pre-op (albumin < 35 g/L)	15 (83.3%)
Albumin^*∗*^	29 (21–40)
Side of empyema (right : left)	1 : 1

^*∗*^Data presented as median (range).

**Table 2:** Patient symptoms.

Symptoms	*n* (%)
Cough	16 (88.9%)
Fever	9 (50%)
Dyspnoea	7 (38.9%)
Chest pain	2 (11.1%)

**Table 3:** Diagnostic yield of mycobacterial smear and culture.

Sample	Smear positive (%)	Culture positive (%)	Smear or culture positive (%)
Sputum	4/17 (23.5%)	7 /17 (41.1%)	7/17 (41.1%)
Intraoperative pleural fluid	2/16 (12.5%)	7/17 (41.1%)	10/17 (58.8%)
Intraoperative pleural tissue	4/16 (25%)	12/16 (75%)	12/16 (75%)
Sputum + intraoperative pleural fluid and tissue	8/17 (44.4%)	16/17 (94.1%)	17/17 (100%)

**Table 4:** Comparison of perioperative and postoperative outcomes.

Outcomes	*n*
*Peri-/postoperative*	
Operation time (mins)^*∗*^	112.5 (55–175)
Chest tube duration (days)^*∗*^	5.5 (3–56)
Hospital Stay (days)^*∗*^	5.5 (3–14)
*Post-operative complications*	
Prolonged air leak of more than 5 days	9 (50%)
Failure of lung reexpansion at 6 months	3 (16.7%)

^*∗*^Presented as median and range.

## Abbreviations

CT: Computed tomography.
